# Stress Granule-Driven Resistance in Cancer: Mechanisms and Emerging Strategies

**DOI:** 10.3390/cancers18020260

**Published:** 2026-01-14

**Authors:** Abirami Rajendiran, Gayathri Ramakrishnan, Takbum Ohn, Aravinth Kumar Jayabalan

**Affiliations:** 1 Texas Tech University Health Sciences Center, El Paso, TX 79911, USA; abirami.rajendiran@ttuhsc.edu; 2 School of Biological Sciences, Department of Cell and Developmental Biology, University of California, San Diego, CA 92093, USA; g1ramakrishnan@ucsd.edu; 3 Department of Cellular and Molecular Medicine, College of Medicine, Chosun University, Gwangju 61452, Republic of Korea; tohn@chosun.ac.kr; 4 Department of Biochemistry and Molecular Biology, Johns Hopkins School of Public Health, Baltimore, MD 21205, USA

**Keywords:** stress granules, biomolecular condensates, drug resistance, metastasis, stress response, cell survival

## Abstract

Stress granules (SGs) are dynamic, membraneless organelles that form in response to stress and play pivotal roles in translational control, RNA metabolism, and cell survival. In cancer, SGs are increasingly recognized as central mediators of therapy resistance, enabling malignant cells to evade apoptosis, reprogram metabolism, and modulate immune responses. Understanding the mechanistic and clinical insights into SG kinetics in healthy versus cancer cells holds significant potential for targeting them in precision oncology. This review integrates current knowledge on how chemotherapeutic agents, oncogenic signaling pathways, and tumor microenvironmental stressors promote SG formation, as well as evidence of altered SG kinetics across tumor types. We further highlight how the upregulation of SG components within the tumor microenvironment shapes cancer cell behavior and adaptability, and how crosstalk between SGs and other biomolecular condensates could contribute to resistance. Finally, we discuss emerging therapeutic strategies targeting SGs, including kinase inhibitors and modulators of SG dynamics, and propose that SGs represent tractable vulnerabilities in precision oncology. By bridging mechanistic insights with clinical implications, this review positions SGs as a promising frontier in overcoming cancer therapy resistance.

## Simple Summary

Environmental and therapeutic stressors constantly challenge cancer cells. To survive, they form transient structures known as stress granules (SGs)—membraneless proteins and RNA clusters that allow cells to temporarily halt normal functions and evade cell death. SGs promote cancer cells’ survival by suppressing apoptosis, reprogramming metabolism, and conferring resistance to chemotherapy. Notably, several anticancer drugs inadvertently induce SG formation, thereby reducing treatment efficacy. Current research aims to inhibit SG formation or promote their disassembly through targeted drugs and molecular interventions, offering promising strategies to overcome therapy resistance in cancer.

## 1. Introduction

The integrated stress response (ISR) is a highly conserved cellular mechanism, present from unicellular organisms to complex multicellular systems, that enables cells to adapt to environmental stress [1]. In healthy cells, a wide range of stressors, including nutrient deprivation, viral infection, oxidative stress, and the accumulation of misfolded proteins in the endoplasmic reticulum (ER), activate ISR—many of which are also characteristic of the tumor microenvironment. Once activated, the ISR provides a transient protective state in which cells downregulate global metabolic and biosynthetic activities to minimize damage. This adaptive pause allows recovery from non-lethal insults and prevents premature activation of apoptotic pathways. Evolutionarily, the ISR represents a conserved survival strategy: it favors adaptation under mild stress while triggering apoptosis when damage becomes irreparable [2]. In cancer, tumor cells may exploit the ISR to endure adverse conditions such as hypoxia and nutrient deprivation [3]. By co-opting the ISR, malignant cells suppress apoptosis, reprogram metabolism, and develop resistance to therapy—making the ISR a promising therapeutic target [4].

The ISR begins when one of four specialized stress-sensing kinases—PKR, PERK, HRI, and GCN2, each of which responds to distinct cellular stressors—is activated. PKR senses viral infection and double-stranded RNA; PERK responds to ER stress and the accumulation of misfolded proteins; HRI detects oxidative stress and heme deficiency; and GCN2 responds to amino acid deprivation [2]. Upon activation, these kinases converge on a common substrate, eukaryotic initiation factor 2 alpha (eIF2α). Phosphorylation of eIF2α inhibits the formation of the ternary complex (eIF2-GTP-tRNAᵢMet), a key component of the translation initiation machinery. This phosphorylation event acts as a rate-limiting step in translation, leading to a global reduction in cap-dependent protein synthesis [5]. Despite this translational repression, selective translation of stress-responsive genes, such as ATF4, continues via cap-independent mechanisms, often mediated by upstream open reading frames (uORFs) that permit reinitiation under stress conditions [6]. This selective translation enables cells to restore cellular homeostasis, mitigate oxidative stress, and reprogram metabolic activities to adapt to stress.

One of the downstream effects of the ISR pathway is the formation of SGs—transient cytoplasmic biomolecular condensates. However, not all ISR conditions lead to SG formation, and not all SGs arise exclusively from ISR activation; their assembly depends mainly on the degree, duration, and nature of the cellular stress encountered [7]. SGs sequester about 400–500 proteins, including apoptotic regulators, and function as dynamic signaling hubs that prioritize cell survival [8,9]. The tumor environment, characterized by diverse and persistent stressors, is inherently more prone to SG formation than non-tumor tissue [10]. Furthermore, growing evidence shows that several anticancer drugs can induce SG formation even at therapeutic concentrations. Consequently, rapid SG assembly during chemotherapy and radiotherapy can inhibit apoptosis, thereby contributing to treatment resistance [11,12,13]. In this review, we outline the biogenesis and broader functions of SGs, their implications in cancer cell survival, and the mechanisms underlying SG assembly—both in response to anticancer drugs and the tumor microenvironment stress. We also discuss emerging therapeutic opportunities targeting SGs. Finally, we highlight key challenges and existing limitations in SG cancer research, including the need to delineate SG versus non-SG functions of core SG proteins for therapeutic targeting.

## 2. Biogenesis, Functions, and Characterization of SGs

**Figure 1:**
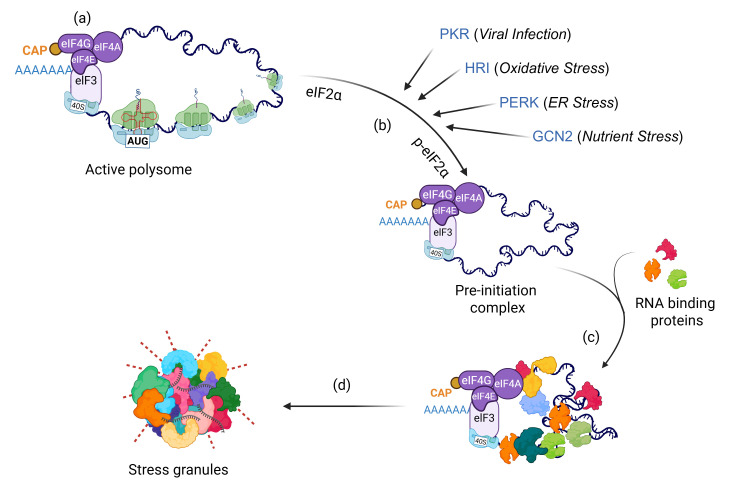
Mechanism of SG assembly. When healthy cells encounter diverse stressors, they rapidly induce global translational arrest via (**a**) p-eIF2α-independent mechanisms such as disruption of the eIF4A complex, or (**b**) phosphorylation of eIF2α by activation of stress kinases (PKR, HRI, PERK, GCN2). This arrest leads to the accumulation of stalled pre-initiation complexes, which interact with (**c**) RNA-binding proteins within minutes and (**d**) assemble into microscopically visible SGs.

When cells encounter unfavorable conditions such as heat shock, oxidative stress, organelle damage, or viral infection, they suppress global translation to conserve energy for recovery [14,15]. Within minutes of stress exposure, translationally arrested messenger ribonucleoprotein (mRNP) complexes condense with RNA-binding proteins, forming microscopically visible, granule-like structures known as SGs (Figure 1). Protein-protein, protein-RNA, and RNA-RNA interactions, together with post-transcriptional and post-translational modifications, finely regulate the dynamics of SGs [16,17,18,19,20,21,22].

Although SG formation typically occurs downstream of the ISR pathway, alternative mechanisms, such as eIF4A inhibition (eIF2α independent) or overexpression of specific SG proteins, can also initiate SG assembly. The RNA-binding proteins G3BP1 and G3BP2 (collectively, G3BPs) are core components essential for SG formation under most stress conditions; simultaneous depletion of both completely abolishes SG assembly [23,24]. SGs can also form independently of G3BP1/2 under certain stress types, such as osmotic stress [24,25]. The composition, size, and dynamics of SGs depend on the type, intensity, and duration of stress, as well as the cell type involved [9,26,27,28,29]. Sodium arsenite induces canonical SGs that recruit most known SG components [30]. In contrast, non-canonical SGs, such as those triggered by selenite, lack eIF3 subunits and several key signaling molecules [9,28,30,31]. SG components mainly include translation machinery (initiation factors, small ribosomal proteins), RNA-binding proteins (G3BPs, TIA1, CAPRIN), post-translational modifiers (PARPs, NEDD8), and mRNAs [9,16,20,32,33]. SG disassembly generally occurs upon stress relief, allowing mRNP complexes to re-enter translation [34].

Although less extensively characterized, SG disassembly also follows stress-specific mechanisms. For instance, autophagy promotes SG clearance under acute stress, whereas ubiquitination plays a prominent role in heat-induced SG turnover [20,35,36]. For detailed insights into the molecular principles governing SG dynamics and composition, we refer readers to recent comprehensive reviews [14,37,38]. Early hypotheses proposed that SGs function as mRNA triage centers, temporarily protecting transcripts during stress or directing them to processing bodies (PBs) for storage, degradation, or re-entry into translation following recovery [39]. Single-molecule imaging has refined this concept, demonstrating bidirectional mRNA exchange between SGs and PBs through transient physical contacts [40]. Recent proteomic analyses reveal that SGs regulate key pathways, including apoptosis, mTOR signaling, and lysosome damage repair, while safeguarding the translation machinery from degradation [8,15,41,42,43]. For example, SGs inhibit cell death by sequestering pro-apoptotic proteins such as RACK1 or caspase-3, thereby preventing MAPK/JNK activation during acute stress [8,44,45,46]. By recruiting DYRK kinase, SGs modulate mTOR signaling and its downstream activation [42,47]. Additionally, SGs accumulate at endolysosomal membrane pores through G3BP1-mediated localization, promoting membrane repair and preventing lysosomal damage-mediated cell death [15,41].

The role of SGs in disease pathogenesis has become increasingly evident from recent studies [48]. During viral infection, antiviral SGs concentrate the innate immune sensor retinoic acid-inducible gene-I (RIG-I), thereby promoting interferon production and amplifying immune signaling [49]. Dysregulated SG dynamics, particularly the persistence of SGs that fail to disassemble, have been implicated in neurodegenerative diseases such as amyotrophic lateral sclerosis (ALS). Mutations in SG-associated nuclear RNA-binding proteins—including hnRNPs, FUS, TIA-1, and TDP-43—lead to cytoplasm mislocalization and the formation of persistent SGs, ultimately disrupting translation and other essential cellular processes [50,51]. Similarly, mutations in the ATPase valosin-containing protein (VCP/p97) impair autophagy-mediated SG clearance and contribute to ALS pathogenesis [35,52]. In cancer, an oncogenic KRAS mutation enhances SG assembly in tumor cells relative to wild-type cells, thereby promoting stress tolerance [10].

SG formation occurs through liquid–liquid phase separation, where mRNA-protein complexes coalesce and phase-separate from the surrounding cytosol. Researchers typically visualize SGs using immunofluorescence or live-cell imaging of established marker proteins such as G3BP1, TIA-1, or eIF3 subunits [53]. These approaches enable direct visualization of SGs at high spatial resolution and the assessment of cell-to-cell variability. Quantitative parameters include SG number per cell, size, total area, and subcellular distribution. Live-cell imaging further permits temporal analysis of SG assembly and disassembly dynamics in response to stress or pharmacological interventions. However, microscopy-based approaches may not readily distinguish bona fide SGs from other cytoplasmic ribonucleoprotein aggregates without careful marker validation. Fluorescence recovery after photobleaching (FRAP) assesses the dynamic properties of SGs by measuring the exchange rates of fluorescently labeled SG-associated proteins within granules. Recovery kinetics provide insights into SG material properties, including liquidity, protein mobility, and maturation state. This approach offers quantitative, kinetic information that static imaging cannot capture and is particularly useful for distinguishing liquid-like SGs from more solid or pathological assemblies. However, photobleaching itself may perturb SG integrity, and recovery measurements can vary depending on the selected marker protein and stress condition [53]. Isolating intact SGs is technically challenging; however, biochemical enrichment of SG cores or proximity-labeling-based proteomic assays has been instrumental in identifying the SG proteome and SG-associated proteins using different molecular baits [9,33,54,55]. Because SGs share numerous components with other cytoplasmic and nuclear condensates—such as PBs, endosomes, and paraspeckles—perturbation studies employing mutants, kinase inhibitors, or small molecules are essential to distinguish SGs from other granular bodies [17,56,57]. A major remaining challenge in the cancer context is the in vivo visualization and characterization of SGs within tumors [58].

## 3. SG Kinetics in Cancer Cells

Early functional studies showed that SGs delay cell death by sequestering signaling molecules involved in apoptosis [7,32]. This phenomenon occurs when cells encounter mild stress. Mechanistically, cells pretreated with SG-inducing agents, such as arsenite, show improved survival when subsequently challenged with apoptotic stimuli, such as etoposide. While etoposide alone induces apoptosis, pre-exposure to arsenite enables cells to sequester key pro-apoptotic proteins such as RACK1 within SGs, thereby inhibiting MAPK-induced apoptotic signaling [8]. Thus, mild stress triggers SG assembly in a cytoprotective manner, providing a temporal buffer that allows cells to determine whether to initiate survival or death pathways. Tumor cells exploit this adaptive mechanism to enhance their survival by transiently forming SGs. In pancreatic cancer, mutant KRAS cells secrete the signaling lipid 15-deoxy-∆12,14-prostaglandin J2 (15d-PGJ_2_), which induces SG formation in neighboring cells via paracrine signaling (Table 1). Remarkably, conditioned media from mutant KRAS cells are sufficient to trigger SG assembly in wild-type cells, conferring chemoresistance [10]. Consistently, both murine models and human pancreatic ductal adenocarcinoma (PDAC) tissues associated with obesity display SGs compared to non-tumor tissues [59].

Recent studies have further refined the understanding that SGs inhibit apoptotic pathways in cancer. The recruitment of executioner caspases, such as caspase-3, into SGs suppresses apoptosis in both normal and malignant cells [46]. Mutant caspase-3 variants that fail to localize to SGs readily execute apoptosis, whereas enforced localization of these mutants to SGs prevents cell death under various stress conditions. In prostate cancer, mutations in the speckle-type POZ (SPOP) enhance SG assembly by stabilizing the RNA-binding protein CAPRIN1 [65]. Studies show that SG formation promotes entry into a quiescence state, confers chemoresistance, and provides a mechanistic link to metastatic latency [64]. In PDAC cells, calcium-dependent phospholipase A_2_ (cPLA_2_)–mediated synthesis of 15d-PGJ_2_ during the G2 phase enhances SG assembly and reveals heterogeneity in SG induction among cancer cell populations [66]. Although early studies regard SGs as sites of translationally stalled mRNAs, evidence from single-molecule imaging and electron microscopy suggests that specific mRNAs continue to undergo translation within SGs [43,67]. Whether this phenomenon represents a general feature of SG biology or is limited to specific subsets of mRNAs, particularly in the context of cancer, remains to be elucidated.

In hepatocellular carcinoma (HCC), *PTEN* mRNA is selectively recruited into SGs, leading to activation of the pentose phosphate pathway and metabolic reprogramming. The serine/threonine kinase RIOK1, upregulated in multiple HCC cell lines, promotes liquid–liquid phase separation via its IDR. RIOK1 interacts with G3BP1 and IGF2BP1, facilitating the sequestration of *PTEN* mRNA within SGs and conferring resistance to tyrosine kinase inhibitors. Consistently, SG-like structures are significantly more abundant in HCC tissues than in healthy liver samples. Notably, the histone deacetylase inhibitor chidamide reverses RIOK1/G3BP1-mediated *PTEN* mRNA puncta formation by downregulating RIOK1 expression and sensitizes HCC cells to the anticancer agent donafenib [62]. Although these reports suggest abnormal SG kinetics in tumor cells, further research is warranted to determine whether tumor cells actively drive these aberrant SG dynamics or whether altered SG formation, in turn, promotes tumor progression and metastasis.

## 4. SG-Mediated Drug Resistance in Cancer Cells

**Figure 2:**
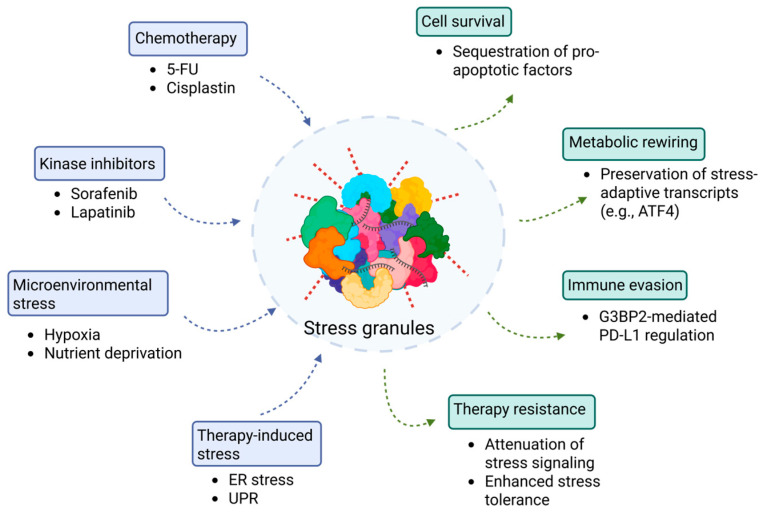
SGs as central regulators of therapy-induced drug resistance in cancer cells. This schematic illustrates how diverse therapeutic and microenvironmental stressors converge to promote SG assembly (blue-boxed components) and drive non-genetic drug resistance in cancer cells (green-boxed components). Upstream triggers—including chemotherapy (5-FU, cisplatin), kinase inhibitors (sorafenib, lapatinib), microenvironmental stress (hypoxia, nutrient deprivation), and therapy-induced stress (ER stress and UPR), leading to eIF2α phosphorylation and translational arrest (blue arrows). This shift promotes SG nucleation, ultimately resulting in cell survival, metabolic rewiring, immune evasion, and therapy resistance (green arrows).

Many cancer therapeutics primarily target kinase activity and cellular stress responses; however, the role of SGs in therapy resistance and their potential as therapeutic targets remain underexplored. SGs contribute to chemoresistance through several mechanisms, primarily by sequestering pro-apoptotic factors such as RACK1, p21(WAF1/CIP1), and EGR1, thereby establishing a transient cytoprotective state [44,68,69,70]. Several anticancer agents induce SGs as a secondary effect, since their concentration or reactive properties activate cellular stress responses that culminate in SG assembly [71]. In certain cancer types, overexpression of oncogenic SG components, such as G3BPs, can trigger spontaneous SG formation, as this process correlates closely with expression levels [72]. Moreover, the tumor microenvironment, enriched with diverse stressors such as hypoxia, nutrient deprivation, and oxidative stress, is inherently predisposed to rapid SG induction compared to non-tumor cells [10,73]. Under these conditions, SG formation inhibits apoptosis-mediated cell death, whereas pharmacologic or genetic modulation of SG assembly sensitizes tumor cells to anticancer drugs (Figure 2).

### 4.1. Induction of SGs by Anticancer Drugs

Numerous anticancer drugs induce SGs in vitro—even at therapeutic concentrations—through canonical pathways involving activation of stress-responsive kinases or through non-canonical mechanisms such as eIF4A inactivation, resulting in both canonical and non-canonical SG assembly [68,74,75]. These transient SGs promote stress adaptation and help cells to avoid apoptosis. Virtually all major classes of anticancer drugs, including multi-kinase inhibitors, DNA damage and alkylating agents, microtubule disruptors, proteasome inhibitors, and translation initiation inhibitors, have been shown to induce SG formation (Table 2) [28,69,71,74]. Notably, certain chemotherapeutic agents are themselves sequestered within SGs, reducing their cytotoxic efficiency [76].

Lapatinib, a tyrosine kinase inhibitor used in the treatment of breast cancer, induces SGs at 20 µM concentration via PERK activation and recruits m^6^A-modified RNAs [75,77]. Inhibition of PERK activity sensitizes T47D breast cancer cells to lapatinib treatment. Interestingly, lapatinib fails to induce SGs in MDA-MB-231 or Hs578T cells, underscoring the cell-type-specific induction of SGs in cancer cells [75]. Sorafenib, used in the treatment of hepatocarcinoma, also triggers SGs through PERK activation in Hep3B cells; however, PERK depletion does not fully prevent SG formation, suggesting that additional signaling pathways are also involved [74].

Bortezomib, a proteasome inhibitor, induces SGs at 1 µM via HRI activation in HeLa cells, HRI depletion blocks SG assembly and increases apoptosis [71]. Similarly, Lomustine, a DNA-alkylating agent, at 200 µM concentration triggers SGs via HRI activation and promotes the recruitment of *Early Growth Response 1 (EGR1)* mRNA into SGs in U2OS cells. *EGR1* encodes a pro-apoptotic transcription factor, and its sequestration within SGs decreases translation, thereby inhibiting cell death and promoting therapy resistance [69]. Microtubule-disrupting agents, such as vinca alkaloids, also induce SG assembly by coordinating the activation of eIF2α and 4E-BP1 signaling pathways [78].

Certain anticancer drugs induce non-canonical SGs or activate SG formation via alternative pathways, such as disruption of mTOR/4EBP1 signaling. Selenite, an experimental anticancer compound, promotes the formation of smaller SGs via 4EBP1-mediated translation arrest. These non-canonical SGs lack several key SG components, including eIF3 subunits [28]. Pateamine A, another experimental compound, induces SG formation independent of eIF2α phosphorylation [79]. Likewise, 5-Fluorouracil promotes SG assembly by incorporating its drug metabolites into RNA, linking nucleotide metabolism to SG biogenesis [68].

**Table 2:** Anticancer drugs that induce SG formation.

Drug Class/Agent	Cancer Cell Line/Model(s)	Mechanism of SG Formation	Functional Consequence(s)	Ref.
Multikinase inhibitor: Sorafenib	Hepatocarcinoma cells	PERK-mediated eIF2α phosphorylation	Formation of canonical SGs	[74]
EGFR/HER2 TKI: Lapatinib	Breast cancer cells	PERK-mediated eIF2α phosphorylation	Formation of canonical SGs	[75]
Vinca alkaloids (Vinblastine/ Vincristine/ Vinorelbine)	Multiple human cancer lines	Dual: PERK-mediated eIF2α phosphorylation and 4E-BP1 activation; cortisone co-treatment activates GCN2	VA-induced SGs lack some signaling components such as RACK1, RSK, TRAF2	[78]
Proteasome inhibitors: (Bortezomib, UPS inhibitors)	Multiple human cancer lines	HRI-mediated eIF2α phosphorylation; GCN2 is implicated with general UPS blockade with MG132	Recruit ARE-rich mRNA into SGs	[71,80]
Antimetabolite: 5-Fluorouracil	Multiple human cancer lines	RNA-incorporation of metabolites lead to eIF2α phosphorylation	Inhibits apoptosis by sequestering RACK1	[68]
DNA-alkylating agent: Lomustine	Human cancer lines	HRI-mediated eIF2α phosphorylation	Selective mRNA partitioning, e.g., EGR1 mRNA	[69]
Platinum-based alkylating agent: Cisplatin	Cochlear cells, Neuroglioma, HeLa	Not entirely on eIF2α phosphorylation; rather by RNA/ribosome damage	Compositionally distinct, small, and persistent SGs	[31,76]
Translation initiation via 4EBP1: Selenite	Cancer- relevant cell lines	Primarily eIF4F suppression via 4E-BP1, with concurrent eIF2α-P	Non-canonical SGs: Altered composition and lack some pro-survival components	[28]
Phytochemical: Morusin	Human cancer cells	PKR-mediated eIF2α phosphorylation	RACK1 sequestration into SGs attenuates caspase-3 activation	[44]

### 4.2. Upregulation of SG-Related Protein Expression in Cancer Patients

Altered protein levels can disrupt cellular homeostasis by driving excessive growth signaling, suppressing apoptosis, promoting stress tolerance, and inducing genomic instability—all of which are key drivers of cancer. Intriguingly, SG assembly can occur spontaneously when SG components, particularly RNA-binding proteins, are overexpressed, even in the absence of external stressors [72]. Expression of multiple SG-related proteins and RNAs is upregulated across diverse cancer types [81]. Although the concentration threshold for each component may vary, these elevated levels are often sufficient to trigger SG assembly, especially under mild stress conditions such as drug exposure or microenvironmental stress [73].

In silico analysis of RNA-seq data from esophageal cancer demonstrates upregulation of SG-associated genes, including *PARPs*, *eIF4E3*, *IGF2BP3*, *OASL*, *PDCD4,* and autophagy-related genes (*DDX58*, *SQSTM1*) following omipalisib treatment—a PI3K/mTOR inhibitor [82]. Omipalisib downregulates p-AKT, p-4EBP1, p-p70S6K, and p-ERK while upregulating FOXO and JAK-STAT signaling pathways [83]. Under these conditions, SG presence promotes epithelial–mesenchymal transition (EMT), immune cell infiltration, and drug tolerance. TWIST1, an essential mechanosensor of EMT, interacts with G3BP2, and loss of G3BP2 drives TWIST1 into the nucleus under conditions of high matrix stiffness in breast cancer cells [63]. *B-cell translocation gene 2 (BTG2)* overexpression can also downregulate TWIST1 expression, producing sustained eIF2α phosphorylation and polysome collapse [84]. In prostate carcinoma PC-3 cells, G3BP1 mediates partitioning of mRNAs into SGs, including pro-apoptotic transcripts such as *BAX*, *BAD*, and *BID*. While arsenite treatment decreases their expression by promoting association with G3BP1 within SGs, G3BP1 knockdown restores protein levels and enhances apoptosis. Significantly, the SG-associated transcriptome varies across cell types (e.g., PC-3, U2OS, HEK293), underscoring the context- and cell-type-specific composition of SGs at the transcriptomic level [85,86].

Analysis of The Cancer Genome Atlas (TCGA) dataset from non-small-cell lung cancer (NSCLC) patients reveals elevated RNA expression of *G3BP1* and *YB1*, which correlates with increased p-AKT levels [87]. Immunohistochemistry (IHC) demonstrates higher G3BP1 expression in both squamous cell carcinoma and adenocarcinoma tissues compared with healthy lung samples. High G3BP1 expression is also associated with poorer survival. Immunoblotting of tumors and adjacent lung tissues from 20 patients shows marked upregulation of G3BP2 and SG-like puncta in these tissues [88]. Overexpression of MG53 (also known as TRIM72), a member of the tripartite motif (TRIM) protein family, modulates G3BP2 activity and reduces tumor volume in a xenograft model by altering G3BP2 distribution. Controlled administration of recombinant human MG53 (rhMG53) further suppresses lung cancer growth when combined with cisplatin treatment [88].

### 4.3. Influence of the Tumor Microenvironment on SG Dynamics

Multiple stressors, including hypoxia, nutrient deprivation, oxidative stress, ER stress, unfolded protein response (UPR), mechanical stress, and metabolic imbalance, define the microenvironment. These conditions can directly induce SGs or precondition cells for rapid SG assembly upon further insult [89]. Under nutrient-deprived conditions, cancer cells shift toward glutamine dependence. SGs fail to form during amino acid starvation; however, glutamine supplementation restores SG assembly. Increased glutamine uptake leads to MYC upregulation and promotes the recruitment of *MYC* mRNA into SGs, potentially protecting it from degradation. Inhibition of MYC or knockout of *G3BP1/2* prevents SG formation and increases apoptosis in MCF-7 cells, but not in non-cancerous MCF10A cells. Moreover, vinblastine inhibits SG fusion and growth under amino acid starvation [61,90]. G3BP2 also modulates tumor-immune interactions by stabilizing the checkpoint protein PD-L1. Inhibiting G3BP2 with C108 reduces PD-L1 expression and increases the infiltration of immune cells into tumors in breast cancer mouse models [91]. As cancer cells metastasize and adapt to these conditions, additional stressors such as chemotherapy or radiotherapy can further trigger SG formation. Repeated exposure to therapeutic regimens may reinforce SG-mediated resistance, posing a major clinical challenge. These findings underscore the need for rational combination therapies that target SG formation to enhance treatment efficacy (discussed in the next section). Together, these microenvironment-driven stresses rewire SG dynamics to protect oncogenic transcripts, reorganize metabolism, and dampen antitumor immunity, thereby creating a cellular state primed for persistent survival and therapy resistance through selective mRNA sequestration, MYC-dependent metabolic buffering, and immune-evasive PD-L1 stabilization.

### 4.4. SGs in Radiotherapy and Immunotherapy

Radiotherapy induces acute oxidative stress, DNA damage, and cytosolic accumulation of nucleic acids, all of which activate ISR kinases and promote SG assembly. SGs formed in response to radiation sequester pro-apoptotic signaling proteins, suppress MAPK/JNK activation, and thereby reduce radiation-induced cell death, contributing to therapy resistance. In addition, radiation-generated cytosolic double-stranded RNA (dsRNA) activates RIG-I and type I interferon (IFN-I) signaling pathways; SGs buffer this dsRNA sensing via G3BP1, thereby dampening IFN-I responses [91,92]. SGs also influence tumor responses to immunotherapy, particularly immune checkpoint inhibitors. SG-associated proteins regulate PD-L1 stability, cytokine translation, and the availability of immunogenic transcripts, thereby shaping the tumor immune phenotype. Moreover, SGs intersect with innate immune sensing: G3BP1 can scaffold RIG-I to enhance IFN-β production but can also sequester dsRNA and limit RIG-I/MDA5 activation, resulting in context-dependent effects on interferon signaling. Because robust IFN-I responses are essential for effective checkpoint blockade, SG-mediated suppression of dsRNA sensing or IFN-I production may reduce immunotherapy efficacy.

### 4.5. Crosstalk Between SGs and Phase-Seperated Compartments

Most studies have traditionally examined the role of SGs in cancer through the lens of their biogenesis; however, emerging evidence shows that SGs also interact with and regulate other phase-separated biomolecular condensates in both the cytoplasm and nucleus. These interactions may profoundly influence cancer cell behavior. One well-studied example is the crosstalk between SGs and PBs. PBs are cytoplasmic condensates that are constitutively present and regulate RNA metabolism, but whose number and size increase under stress conditions. SGs and PBs physically dock and exchange mRNAs, which may subsequently be stored, degraded, or redirected for translation reinitiation once the stress subsides. Given that cancer cells frequently alter the SG proteome and transcriptome, it is critical to determine how these tumor-specific changes affect mRNA exchange dynamics and downstream RNA metabolism via PBs [14,93,94].

Beyond PBs, SGs also regulate several other biomolecular condensates relevant to tumor biology. In the nucleus, SGs modulate paraspeckle formation by sequestering negative regulators such as UBAP2L, thereby relieving their inhibitory effects on paraspeckles [95,96]. Similarly, by sequestering ribosomal proteins, transcription factors, and RNAs, SGs influence nucleoli function and may alter transcriptional programs [97,98]. SGs also interact with multiple membrane-bound organelles—including ER, autophagosomes, mitochondria, lysosomes, and exosomes—in a context-dependent manner. For example, during the UPR, SGs associate closely with the ER and modulate mitochondrial signaling and apoptosis [99]. SG clearance is primarily mediated by autophagy, linking SG dynamics to the broader proteostasis network [36,100,101]. Exosomes share approximately 29% of SG components, and the SG-associated RNA-binding protein YB-1 recruits miR-223, a key regulator of apoptosis [102].

In addition to their interaction with membrane-less and membrane-bound organelles, SGs regulate several key signaling pathways, including ISR, mTOR signaling, RNA metabolism, NF-kB signaling, MAPK signaling, and apoptosis [8,103,104,105]. Because each of these pathways independently contribute to tumor growth, survival, and metastasis, SG-mediated modulation of their activity in cancer cells warrants further investigation. Future studies are needed to elucidate how SGs crosstalk with organelles and signaling networks collectively contribute to oncogenesis and therapy resistance. By coordinating RNA storage, degradation, and signaling through interactions with other condensates and organelles, SGs create a pro-survival network that enhances cancer cell plasticity and treatment resistance.

## 5. Therapeutic Options Targeting SGs

**Figure 3:**
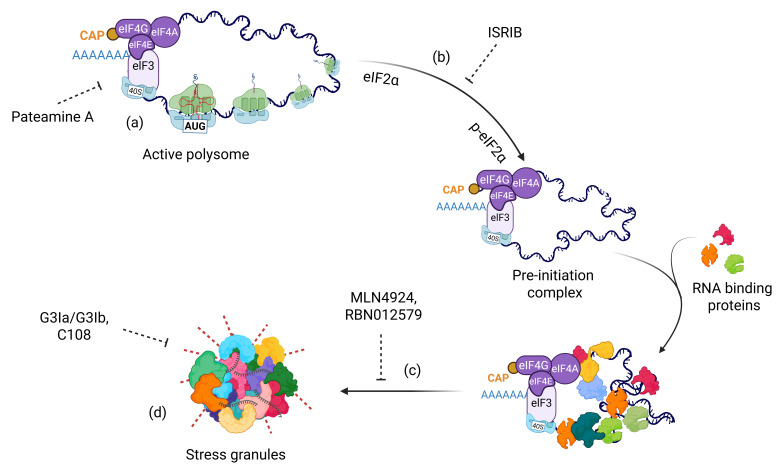
Therapeutic strategies targeting distinct stages of SG assembly. (**a**) Translation inhibitors such as Pateamine A that disrupt global translation; (**b**) Inhibition of stress kinase activation (e.g., PERK, HRI, GCN2, and PKR) using ISRIB; (**c**) Targeting post-translational modifications such as NEDDylation or ADP-ribosylation using MLN4924 and RBN012579, respectively; and (**d**) Modulators of biomolecular condensates, including small molecules that target SG core components and key redox reactions.

Although extensive research using animal models or patient-derived induced pluripotent stem cells (iPSCs) is still needed to define the critical role of SGs in drug resistance, in vitro studies have clearly demonstrated that SGs enable cancer cells to evade cell death [8,106]. Therefore, SGs represent a rapidly reversible, non-genetic survival strategy that cancer cells exploit to withstand chemotherapy, targeted inhibitors, and microenvironmental stress. Because SGs suppress apoptosis, preserve pro-survival transcripts, and buffer cells against proteotoxic and metabolic stress, therapeutically disrupting SG assembly or accelerating their clearance can convert transient drug tolerance into prolonged tumor control and delayed disease recurrence. Importantly, multiple SG-targeting strategies, including PTM inhibitors, biomolecular condensate modulators, stress-kinase blockers, and indirect metabolic interventions, have been shown to enhance the cytotoxicity of standard chemotherapeutics in preclinical models. These findings underscore a key translational principle: SG-directed agents improve the efficacy of existing therapies by disrupting the stress-adaptive mechanisms that enable cancer cells to survive treatment. As such, rational combination regimens that pair SG inhibition with chemotherapy, kinase inhibitors, or immunotherapy hold substantial promise for overcoming drug resistance and improving clinical outcomes (Figure 3).

### 5.1. Post-Translational Modifications

Post-translational modifications (PTMs) of proteins play crucial roles at multiple stages of SG dynamics, particularly during condensation, and nearly all major PTM types are involved in this process (Figure 1) [16,17,18,19,20,107,108,109]. Targeting PTMs offers two significant advantages: (1) the core protein function can remain intact while specifically inhibiting SG-associated PTMs, and (2) several anticancer drugs that modulate PTMs have also been found to inhibit SG formation. For example, MLN4924, a drug used in breast cancer therapy, suppresses SG assembly by targeting the NEDDylation pathway [18,110]. NEDDylation of splicing factor SRSF3, ribosomal proteins, and other SG components enables their localization within SGs. Mechanistically, NEDDylated SRSF3 interacts with other components (e.g., TIA1) and facilitates proper SG assembly. In addition, NEDDylation modifies Cullin proteins, key elements in the p53 regulatory pathway [111]. MLN4924 thus has the dual potential to sensitize breast cancer cells while simultaneously inhibiting SG formation. Further studies must determine this phenomenon in vivo and assess whether this drug can be repurposed for additional cancer types (Figure 3).

Another prominent PTM involved in SG regulation is ADP-ribosylation—a nucleic acid-like modification that plays critical roles in various cellular processes [112,113]. ADP-ribosyl transferases, collectively known as poly(ADP-ribose) polymerases (PARPs), mediate ADP-ribosylation by modifying specific target proteins [114]. Targeting nuclear PARPs in *BRCA1/2*-mutant breast cancer is an established anticancer therapy [115]. Recent studies, however, have identified cytoplasmic PARPs as key modulators of SG assembly [16,109,113,116,117]. Notably, the contribution of individual PARP enzymes to SG formation appears to be cell-type specific [118]. For example, PARP14 is primarily implicated in ovarian cancer cells, whereas both PARP10 and PARP14 contribute to SG formation in osteosarcoma cell lines [109,116]. Inhibition of PARP14 activity with RBN012579 sensitizes ovarian cancer cells to thapsigargin treatment and reduces tumor growth in OVCAR3 xenografts [116,119]. Given the availability of selective inhibitors against individual PARP enzymes, further studies must delineate which specific PARP(s) drive SG formation across cancer types and determine whether targeting these enzymes can enhance therapeutic sensitivity [120].

### 5.2. Biomolecular Condensate Modulators

Modulating liquid–liquid phase behavior is a promising, rapidly evolving approach for targeting diseases associated with aberrant biomolecular condensates [121]. Several small-molecule modulators already demonstrate effective SG inhibition in both in vitro and in vivo models [122,123]. Significantly, these compounds enhance the efficacy of anticancer therapies when co-administered with conventional chemotherapeutic agents, highlighting their potential as adjunctive SG-targeted therapeutics (Table 3). Direct modulators of G3BP1/2 condensation—such as G3Ia/G3Ib—along with G3BP1 degradation via PROteolysis TArgeting Chimera (PROTAC) systems and inhibition of G3BP2 activity through specific binders like C108 or rhMG53, have all demonstrated effective suppression of SG formation while improving drug sensitivity (Figure 3). For instance, SG core-derived peptides inhibit SG assembly and enhance sorafenib responsiveness when used in combination treatment [124]. Beyond targeting core SG components, compounds that modulate the condensation behavior by targeting intrinsically disordered proteins (IDPs) also hold promise, potentially mitigating SG formation without impairing protein function [123].

### 5.3. Blocking Activation of Stress Kinases

In normal cells, stress-responsive kinases such as PERK, PKR, GCN2, p38, and JNK trigger transient translational repression through eIF2α phosphorylation, leading to short-lived SG formation. Oncogene signaling, oxidative stress, hypoxia, and nutrient deprivation chronically or repeatedly activate these same kinases in cancer cells, driving persistent or recurrent SG assembly that tumor cells actively exploit. Cancer-associated SGs selectively sequester pro-apoptotic factors and mRNAs encoding cell-cycle inhibitors, while allowing continued translation of survival, DNA repair, and drug resistance programs. Thus, inhibition of stress kinase activation represents an alternative strategy to block SG formation (Figure 3) [126]. Knocking down or pharmacologically inhibiting stress kinases, HRI, GCN2, PERK, and PKR, sensitizes cancer cells to therapeutic agents. For example, HRI knockdown increases sensitivity to bortezomib treatment, PERK depletion enhances sorafenib-induced cell death, and G3BP1 knockdown sensitizes cells to morusin treatment [44,80,124]. A small-molecule screen for potential SG inhibitors identified β-estradiol, progesterone, and stanolone (EPS), which efficiently inhibit PKR-dependent SG formation in HeLa cells. Combination treatment with EPS and cisplatin (CDDP) or EPS and paclitaxel markedly reduces drug resistance under hypoxic conditions in HeLa cells [127]. This effect appears to be drug- and cell-type specific: SGs still form in MCF-7 cells following arsenite or heat-shock exposure. Consistently, eIF2α phosphorylation levels are reduced in HeLa cells during EPS treatment but remain unchanged in MCF-7 cells. Moreover, EPS treatment fails to inhibit SG formation in other cancer cell lines, including human colon (HCT116), breast (MDA- MB-468), pancreatic (PANC-1), bladder (5637, RT4), and ovarian (OVCAR-5) cells, further corroborating the cell type-specific nature of SG induction across cancer types.

### 5.4. Indirect Modulators of SG Dynamics

SGs can also be targeted indirectly by modulating metabolic and stress-sensing pathways or by enhancing SG clearance. One such approach involves using glutaminase inhibitors to modulate SG assembly in KRAS-driven therapeutic resistance. Oncogenic KRAS upregulates NRF2, a master regulator of oxidative stress responses, thereby promoting metabolic rewiring and increased glutamine dependence, which contributes to resistance to gemcitabine treatment [90]. Glutamine deprivation or pharmacologic inhibition of glutaminase attenuates SG formation under these conditions, disrupts redox homeostasis, and sensitizes cancer cells to gemcitabine. Clinically approved autophagy activators, such as rapamycin or trehalose, can also enhance SG clearance; however, determining their effectiveness in modulating SGs and inhibiting therapy resistance remains a robust area of investigation.

## 6. G3BP1 and G3BP2: Beyond SG Formation

While upregulated expression of core SG components such as G3BP1 and G3BP2 can lead to spontaneous SG formation, it is essential to recognize that these proteins perform diverse cellular functions that may also be altered in cancer [128]. Beyond their role in SG nucleation, G3BPs participate in RNA metabolism, signal transduction, cell proliferation, immune regulation, and neuronal development [45]. A substantial fraction of G3BPs remains outside SGs, and how these cytoplasmic pools contribute to non-SG-related functions in cancer remains poorly understood [54].

For instance, G3BP1 tethers the tuberous sclerosis complex (TSC) to lysosomes, thereby regulating mTOR signaling and lysophagy [129,130]. In primary hepatocytes, the RNA-binding protein clustered mitochondria homolog (CLUH) forms ribonucleoprotein assemblies that control mitochondrial biogenesis. During starvation, CLUH granules recruit G3BP1/2 along with mTOR to coordinate mitophagy [131]. G3BP1 is specifically required in pancreatic β-cells to regulate insulin secretion, whereas G3BP2 also exhibits distinct regulatory roles [132]. Arginine methylation of G3BP2 modulates low-density lipoprotein receptor-related protein 6 (LRP6) phosphorylation and Wnt/β-catenin signaling [133]. During viral infection, G3BPs exhibit both proviral and antiviral activities depending on the virus type [134]. Although current evidence does not conclusively indicate that G3BPs negatively regulate cancer progression, their multifunctional nature suggests such possibilities. G3BP1 can degrade mRNAs with highly structured 3’ untranslated regions while stabilizing and promoting the translation of a subset of short, stable, and highly expressed transcripts [135,136]. The functional roles of these preferentially bound transcripts—and their translation status—remain unexplored in cancer contexts. It is critical to employ robust experimental approaches, including functional and mutational assays, targeted protein degradation, and live-cell imaging, to distinguish SG-specific versus non-SG-related functions of G3BPs. Overall, the multifaceted functions of G3BPs extend far beyond SG formation, underscoring the need to determine their SG-dependent effects from their broader cellular roles to better understand their contribution to cancer biology.

## 7. Challenges/Outstanding Questions

Challenges in understanding SG composition: SG kinetics vary markedly across cell types and with the intensity or duration of stress encountered by healthy cells [9,27,137]. In cancer cells, altered RNA and protein expression profiles can further modify the SG proteome and transcriptome. For instance, HeLa cells form SGs with distinct kinetics in response to different stressors, and the resulting SG proteomic landscape varies according to both cell type and stress type [7,9]. Within the tumor microenvironment, differential expression of specific proteins or mRNAs may give rise to compositionally distinct canonical or non-canonical SGs. Identifying these compositional differences across cancer types is essential for designing effective therapeutics targeting SGs. However, one significant challenge in the SG field is defining the “complete” SG composition, as specific proteins and mRNAs continuously exchange between SGs and the cytosol. Thus, a fundamental question remains: does the experimentally identified SG proteome truly represent the full complement of SG components, or only a dynamic subset captured under specific conditions?

Need of in vivo models in SG characterization: While studies of SGs in tissue culture systems offer experimental control through precise drug treatments and imaging or biochemical assays, in vivo models are essential to advance SG research in cancer. Such models are critical for recapitulating the complexity of the tumor microenvironment. Although SG composition is known to vary across cancer types in vitro, it remains unclear whether these differences persist in vivo. Because the tumor microenvironment is continuously exposed to diverse and fluctuating stress conditions, it remains unknown whether the SG proteome dynamically changes over time under these conditions. A key experimental challenge will be selecting the appropriate SG marker(s) and optimizing the timeline for immunohistochemistry (IHC) to capture SG dynamics in tumor tissues accurately.

Are SGs a byproduct of cancer cells? SGs protect cells from apoptosis by transiently sequestering pro-apoptotic factors and providing a temporary survival advantage. These dynamic structures typically disassemble once the stress is resolved, even under prolonged exposure. Whether this transient SG presence reprograms cancer cells or merely represents a byproduct of cellular stress remains an open question. Nevertheless, targeting oncogenic G3BPs through small-molecule modulators represents a promising strategy to overcome therapy resistance, as such interventions could simultaneously inhibit both SG-dependent and SG-independent oncogenic functions.

Authentication of SGs in existing model systems: Applying orthogonal approaches, including FRAP, live-cell imaging, perturbing condensate formation, and using mutant constructs, is essential to determine whether the observed condensates are bona fide SGs. However, validating SG identity in vivo remains a significant challenge. For instance, diet-induced obese mice exhibit elevated SG levels compared with mice fed a standard diet, as indicated by G3BP1 staining [59]. Yet, a recent study challenged this observation, reporting that G3BP1 puncta were predominantly perinuclear rather than evenly distributed throughout the cytoplasm [58]. Intriguingly, that study also found that the RNA expression of several SG-associated components was significantly enriched in pancreatic tissue [58]. This observation raises the question of whether the observed puncta truly represent SGs or instead correspond to G3BP1 aggregates, given that G3BP1 possesses intrinsic dimerization propensity and the ability to form RNA-containing condensates.

## 8. Discussion

SGs have emerged as pivotal regulators of cellular stress adaptation in cancer biology. By modulating global translation, sequestering pro-apoptotic factors, and reprogramming RNA metabolism, SGs enable malignant cells to withstand both intrinsic stressors within the tumor microenvironment and extrinsic therapeutic pressures. Although in vitro studies have generated much of the mechanistic insight, patient-derived samples underscore the clinical relevance of SGs. Elevated expression of SG-associated proteins, such as G3BP1, G3BP2, IGF2BP3, and YB1, correlates with poor prognosis and reduced survival in lung, pancreatic, and breast cancers [87]. These observations highlight critical clinical implications, as SG-forming capacity could serve as a predictive biomarker of therapy resistance, reflecting enhanced cellular ability to adapt to translational stress induced by anticancer treatments. Accordingly, assessment of SG protein expression may help identify tumors with increased stress tolerance. Moreover, SG components are useful for stratifying patients in emerging SG-targeted therapeutic strategies, enriching clinical trials for tumors most dependent on SG-mediated stress adaptation. Finally, changes in SG assembly or localization may function as pharmacodynamic biomarkers, providing a readout of target engagement and therapeutic efficacy. Importantly, IHC detection of SG-like puncta in tumor tissues supports the view that SGs are not merely cell culture artifacts but represent biologically and clinically significant condensates. Together, these applications highlight SGs as actionable, non-genetic vulnerabilities in cancer.

SG heterogeneity across cancer types remains an underexplored yet critical gap in current research. Solid tumors, often exposed to hypoxia and nutrient deprivation, may exploit SG-mediated suppression of apoptosis to enhance survival. In contrast, hematological malignancies—subject to distinct metabolic and immune pressures—may engage SGs via alternative regulatory mechanisms. Notably, the SG transcriptome and proteome exhibit substantial variability across cell lines and patient-derived tumors, suggesting that SG-driven resistance is highly context-dependent. This heterogeneity represents both a challenge and an opportunity: SG composition or elevated expression of specific SG proteins could serve as tumor-specific biomarkers to guide therapeutic design. An emerging frontier in SG research is their role in modulating the tumor–immune interface. SGs may attenuate antitumor immunity by altering cytokine signaling, regulating RNA-sensing pathways, or sequestering immune-related transcripts [92]. These immunomodulatory functions suggest that SGs may contribute not only to resistance against chemotherapy and radiotherapy but also to immune checkpoint blockade failure—an area that warrants extensive future investigation.

Therapeutic studies have identified several vulnerabilities within SG biology. Promising strategies include combinational therapies targeting stress-responsive kinases (e.g., PERK, HRI, GCN2, PKR), modulating post-translational modifications such as NEDDylation, SUMOylation, and ADP-ribosylation, and disrupting aberrant biomolecular condensate formation. Small molecules that interfere with pathological phase separation—such as lipoamide derivatives and G3BP1/2 modulators—or that enhance SG clearance through autophagy represent exciting avenues for intervention. Given that SG-associated proteins such as G3BP1 and G3BP2 also perform SG-independent roles in RNA metabolism and signal transduction, it is essential to delineate condensate-specific from basal cellular functions to minimize off-target effects and optimize therapeutic precision.

## 9. Conclusions

SGs embody a dual role in cancer biology—providing transient cytoprotection under mild stress while being co-opted by tumors to evade therapy-induced apoptosis. As research progresses from descriptive studies to translational applications, the integration of transcriptomic, proteomic, and patient-derived datasets continues to illuminate the clinical significance of SGs. Moving forward, the field must prioritize the development of robust biomarkers to monitor SG activity within tumors, advanced imaging modalities to visualize condensates in vivo, and the design of clinically viable SG-specific inhibitors. Embedding SG biology within the broader framework of drug resistance mechanisms will enhance our understanding of tumor adaptability and therapeutic resilience. With precise molecule targeting, SGs hold the potential to transition from indicators of poor prognosis to actionable therapeutic vulnerabilities—paving the way for innovative strategies in precision oncology.

## Figures and Tables

**Table 1:** Key signaling pathways that drive SG formation in cancer cells.

Signaling Pathway	Primary Trigger	Mechanism of SG Formation	Functional Consequence(s) in Cancer	Ref.
Oncogenic KRAS signaling	KRAS mutations, paracrine 15d-PGJ_2_ secretion	Lipid-mediated stress signaling enhances SG nucleation	Rapid SG assembly, chemoresistance, paracrine SG induction	[10,60]
MYC-driven metabolic rewiring	Glutamine addiction, nutrient stress	MYC upregulation promotes SG formation; MYC mRNA recruited into SGs	Selective protection of oncogenic transcripts; apoptosis resistance	[61]
RIOK1–IDR–LLPS pathway	RIOK1 overexpression in HCC	IDR-mediated phase separation with G3BP1/IGF2BP1; PTEN mRNA sequestration	PPP activation, resistance to TKIs	[62]
UPR–ER–mitochondrial stress crosstalk	Hypoxia, ROS, ER stress	SGs form adjacent to the ER; modulate mitochondrial apoptotic signaling	Prevents MOMP; enhances survival	[60]
Tumor microenvironmental stress	Hypoxia, acidity, mechanical stress, and nutrient deprivation	Multimodal ISR activation resulted in altered SG kinetics	EMT, immune evasion, quiescence, drug tolerance	[63,64]

MOMP—Mitochondrial outer membrane permeabilization; UPR—Unfolded protein response; EMT—Epithelial-to-mesenchymal transition; PPP—Pentose phosphatase pathway; TKI—Tyrosine kinase inhibitors; IDR—Intrinsically disordered regions.

**Table 3:** Inhibitors/modulators that affect SG kinetics.

Inhibitors/ Modulator	Targeting Mechanism	Effect on SGs	In Vivo/ Tumor Study	Clinical Implications	Ref.
G3Ia/G3Ib	Interacts with NTF2-like domain and disrupts condensation	Prevents/dissolves pre-formed SGs	None reported		[122]
C108	Binds with G3BP2	Suppresses SG formation	In tumor bearing mice, C108 improves survival and promotes CD8 T-cell infiltration	Preclinical	[91]
PROTAC	Degrades G3BP1	Prevents/dissolves pre-formed SGs	Reduces tumor growth in vivo, suppresses fibroblast-mediated cancer growth	Preclinical	[125]
Lipoamide	Redox modulation of IDPs which shift the balance away from condensation	Prevents/dissolves Pre-formed SGs	None reported	Phase II	[123]
ISRIB	Enhances eIF2B activity and counteracts the effect of p-eIF2α	Prevents/dissolves Pre-formed SGs	Improve paclitaxel- resistant tumors (PDX574) treatment	Preclinical	[126]

G3Ia/G3Ib—G3BP inhibitor a and b; NTF2—Nuclear transport factor 2; IDPs—Intrinsically disordered proteins; PROTAC—Proteolysis targeting chimera; ISRIB—Integrated stress response inhibitor; PDX574—patient-derived xenograft.

## Data Availability

No new data were created or analyzed in this study.
